# Prediction of potential distribution areas and priority protected areas of *Agastache rugosa* based on Maxent model and Marxan model

**DOI:** 10.3389/fpls.2023.1200796

**Published:** 2023-07-24

**Authors:** Yongji Wang, Ruxia Zhao, Xueyong Zhou, Xiaolong Zhang, Guanghua Zhao, Fenguo Zhang

**Affiliations:** ^1^ School of Life Science, Shanxi Engineering Research Center of Microbial Application Technologies, Shanxi Normal University, Taiyuan, Shanxi, China; ^2^ School of Resources and Environment, Shanxi University of Finance and Economics, Taiyuan, Shanxi, China; ^3^ Administrative Office, Shanwei Middle School, Shanwei, China

**Keywords:** *Agastache rugosa*, Marxan model, climate change, prediction of suitable area, ecospat package, environmental factor

## Abstract

*Agastache rugosa* (Fisch. & C. A. Mey.) Kuntze has been widely studied because of its high medicinal and edible value. Establishing the priority protected area of wild *A. rugosa* can provide scientific basis for the protection of germplasm resources. In this study, we predicted the potential suitability distribution area of *A. rugosa* under the current and future climate scenarios with the MaxEnt model, and the dominant climate factors affecting the distribution of *A. rugosa* were analyzed. Based on the above results, we predicted the priority protected areas of *A. rugosa* with the Marxan model. The results showed that *A. rugosa* is mainly distributed in the eastern and central regions of China at present. In future, the suitable area of *A. rugosa* will increase, otherwise a few areas will shrink back and migrate to the high latitude areas as a whole. Hydrothermal conditions are the main environmental factors affecting the distribution of *A. rugosa*. The priority protected areas of *A. rugosa* are mainly distributed in Chongqing, eastern Sichuan, southern Guizhou, western Hunan and Hubei and southwestern Shaanxi, which are basically consistent with the highly suitable areas predicted by Maxent model. The results of this study are of great significance for the protection and rational utilization of species of Agastache.

## 1 Introduction

Climate change affects the growth and development, geographical distribution and population size of plants (Pounds et al., 2006), among which climate change has the most obvious influence on plant distribution. With global warming, the concentration of carbon dioxide gradually rises, which promotes the distribution of plants to move to higher latitudes. However, climate change has different effects on different plants, so studying plant responses to climate change is important for biodiversity conservation. Maximum entropy model (Maxent) is a model based on the principle of niche. It can analyze and predict the potential geographical distribution pattern of species by fitting the probability distribution with the maximum entropy value using the information on species distribution position and environmental variable data (Xu et al., 2015), and it is a method to obtain the potential geographical distribution of species accurately and quickly (Li et al., 2019; Sun et al., 2019). Compared with other models for predicting species distribution, such as niche factor model (ENFA), bioclimate analysis system (BIOCLIM) and genetic algorithm model of rule set (GRAP), the maximum entropy model (Maxent) has higher modeling accuracy (Phillips and Dudik, 2008; Zhu et al., 2022), and can achieve better prediction results even in the case of lack of species distribution coordinates (Liu et al., 2018).

Niche theory mainly reveals the interaction between species, organisms and environment, including niche overlap theory and niche width theory. Niche overlap mainly quantifies the overlapping utilization of resources by species, revealing the utilization and competition of resources and environment by biological communities; The niche width can reflect the range of resources dominated by biological communities (Li et al., 2014; Hu et al., 2021). Ecospat package is used for spatial ecological analysis, especially for species distribution, niche and community construction. ENMTools can analyze species niche evolution and calculate niche width. Using niche theory, the niche overlap index and niche width are quantified. By analyzing the differences of niche overlap and niche width of a species in different periods and different climatic backgrounds, the niche characteristics of the species in different climatic conditions are explored, and the accuracy of the Maxent model in predicting the potential distribution of the species is verified.

Identification and division of priority areas is an important means to protect biodiversity (Richard et al., 2004; Mou et al., 2021), and one of its purposes is to apply the limited biological protection resources to the areas most worthy of protection, so as to achieve the optimal protection effect (David and Eric, 1998; Myers et al., 2000). Delineation of priority protected areas should consider practical operability and feasibility, such as capital, land, manpower and other restrictive factors in building a protection system (Zhang et al., 2011). From the choice of model tools, Marxan model can repeatedly and randomly select a certain number of planning units, can spend the least grid of protected areas to establish a protection scheme (Zhao, 2021), and scientifically and reasonably delimit biological priority protected areas from the perspective of biodiversity, so it has strong application and operability.

*A. rugosa* is a herbaceous species from the family Lamiaceae. It is used as a whole herb for medicine, with the functions of relieving fever, eliminating dampness and invigorating stomach, and having the effect of preventing and treating epidemic diseases (Shen and Zhang, 2023). In addition, it is the raw material for manufacturing a variety of Chinese patent medicines, and also has been announced by the National Health and Health Commission as a medicinal and edible homologous plant (Que et al., 2017; Fan et al., 2021), which has high medicinal and edible value. It is distributed all over the north and south of China and has a long cultivation history. It is mainly found in Jiangxi, Guangdong, Sichuan, Jiangsu, Zhejiang, Hunan and other provinces (Chen, 2017). Collecting *A. rugosa* from the wild can lead to the decrease of local wild population, which is not conducive to the breeding plan and the protection of seed gene bank. However, the research on the potential distribution prediction and priority protected area planning of *A. rugosa* under current climate and future climate change scenarios has not been reported. Therefore, the study aimed to (1) predict the potential distribution areas of *A. rugosa* in China and compare the spatial distribution pattern under different climate scenarios using the Maxent model and ArcGIS V10.5 software, (2) analyze the main environmental factors affecting the geographical distribution of *A. rugosa*, (3) analyze niche difference and verify the accuracy of the model with the ecospat package, (4) delimit priority protected areas and areas highly helpful to the growth of *A. rugosa* with the Marxan model. The results could reduce the wild collection of this species and provide scientific basis for the protection of wild *A. rugosa* and its germplasm resources.

## 2 Materials and methods

### 2.1 Data source and processing

#### 2.1.1 Data source and processing of distribution points of *A. rugosa*

**Figure 1:**
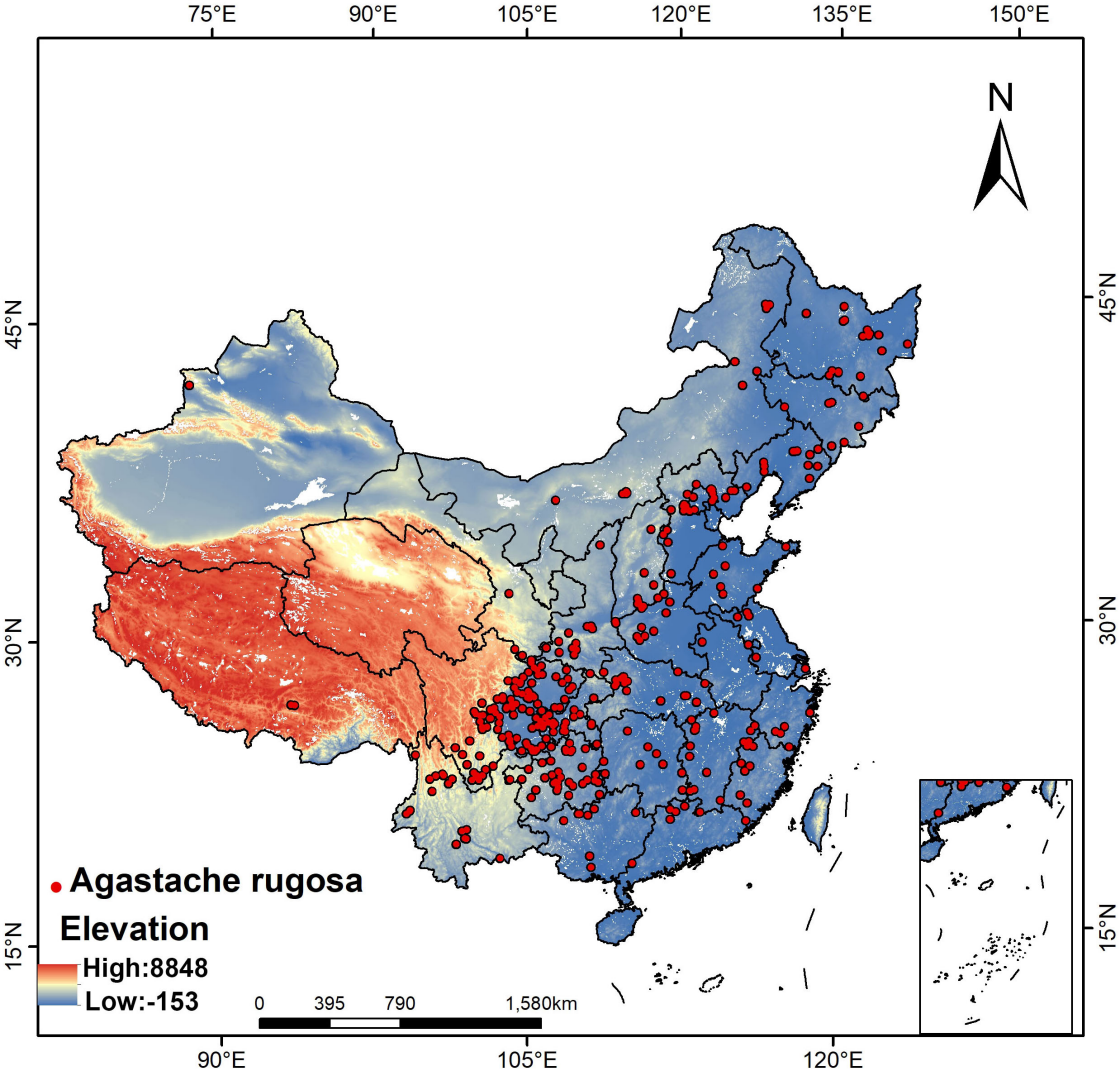
Distribution position of sampling points of *A. rugosa*.

By sorting out the information on *A. rugosa* recorded in China Digital Herbarium (CVH, http://www.cvh.ac.cn/) and combining with the distribution point data onto *A. rugosa* in the Global Biodiversity Information Platform (GBIF, https://www.gbif.org/), the existing distribution position of *A. rugosa* is preliminarily obtained, and then the corresponding latitude and longitude coordinates of each distribution point are obtained by Baidu coordinate picking system(https://api.map.baidu.com/lbsapi/getpoint/). In order to reduce the error caused by cluster effect of the modeling process, a series of repeated and wrong samples were deleted by R package, and finally 390 distribution points of *A. rugosa* were obtained. As shown in **Figure 1**.

#### 2.1.2 Data acquisition and screening of environmental factors

The study used 37 environmental factors, including 19 bioclimatic factors (annual mean temperature, mean diurnal range, isothermality, temperature seasonality, max temperature of warmest month, min temperature of coldest month, temperature annual range, mean temperature of wettest quarter, mean temperature of driest quarter, mean temperature of warmest quarter, mean temperature of coldest quarter, annual precipitation, precipitation of wettest month, precipitation of driest month, precipitation seasonality, precipitation of wettest quarter, precipitation of driest quarter, precipitation of warmest quarter, precipitation of coldest quarter), 2 topographic factors (altitude, slope), 16 soil factors (basic saturation, carbonate or lime content, sulfate content, cation exchange capacity of cohesive soil, cation exchange capacity of soil, clay content, volume percentage of crushed stone, exchangeable sodium salt, conductivity, organic carbon content, pH, soil bulk density, sand content, silt content, classification of exchangeable base, USDA soil texture). The current (1970-2000) and future (2050s:2041-2060, 2090s:2081-2100) climate data are all derived from the world climate database Worldclim2.1 (http://www.worldclim.org/), and each period includes 19 climate factors bio1-bio19. The future climate data will be selected in the sixth phase of a new round of international coupled model comparison plan (CMIP6), which is different from the typical concentration path (RCPs) scenario in CMIP5, and is a combination scenario of different shared socio-economic paths (SSPs) and RCPs, and contains the meaning of future socio-economic development (Jiang et al., 2020). In this study, three combined scenarios, SSP1-2.6, SSP2-4.5 and SSP5-8.5, were selected, which respectively represented low radiation forcing scenario, medium radiation forcing scenario and high radiation forcing scenario. The spatial resolution of data is 2.5 arc-minutes (~5km). The soil factors are derived from the soil data set based on the World Soil Database (HWSD) provided by the Science Data Center of Cold and Arid Regions, and the topographic data are derived from the geospatial data cloud (http://www.gscloud.cn/). In this study, the suitable area prediction was made under the assumption that soil factors and topographic factors would not change in the next 70 years (Zhang et al., 2018).

Based on the 37 environmental factors mentioned above, we considered the importance of variables obtained by the jackknife technique and quantitatively evaluated the impact of environmental factors on the geographic distribution of *A. rugosa*, using pearson and VIF to check the correlation and importance of environmental factors. Spearman correlation analysis and multicollinear VIF variance expansion factor analysis were performed on the point interpolation data in R language, and the environmental factors with correlations less than 0.7 and VIF variance expansion factor values less than 5 were initially screened. VIF variance expansion factor is also called the reciprocal of tolerance. When VIF<5, there is no multicollinearity among factors; when 10<VIF<100, there is multicollinearity among factors; when 100<VIF, there is serious interfactor multicollinearity.

Jackknife is a resampling method, and its original motivation is to “reduce the bias of estimation”. Specifically, for the population with unknown distribution, samples with sample size of n are extracted from it. Using sample statistics $\theta_{n}$ to estimate the overall parameter θ will produce some errors, especially in the case of small samples. In order to solve such a problem, the statistic calculated after cutting off the I-th individual from the original sample can be recorded as $\theta_{-i}$ . Generally speaking, there will be a constant (deviation) and an infinitesimal difference between the estimated value and the actual value:


$$E\left(\theta_{n}\right)=\theta +\frac{a}{n}+\frac{\epsilon }{n^{2}}$$

$$E\left(\theta_{-i}\right)=\theta +\frac{a}{n-1}+\frac{\epsilon }{{\left(n-1\right)}^{2}}$$

The difference between $n\theta_{n}$ and $\left(n-1\right)\theta_{n}$ is defined as cutting off the first Virtual value $\theta^{,}\theta_{\;i}$ after 2 individuals:


$$\theta^{,}{\;}_{-i}=n\theta_{n}-\left(n-1\right)\theta_{-i}$$

$$E\left(\theta^{,}{\;}_{-i}\right)=nE\left(\theta_{n}\right)-\left(n-1\right)E\left(\theta_{-i}\right)=\theta +\frac{∊}{n}-\frac{\epsilon }{n-1}=\theta -\frac{\epsilon }{n\left(n-1\right)}$$

The expected value of the virtual value is equal to the overall parameter minus an infinitesimal amount, which shows that its estimation of the overall parameter compared with $\theta_{n}$ is more accurate. Therefore, the mean value of the virtual value can be used as an unbiased estimate of the overall parameters: (Lian et al., 2018).


$${\bar{\theta }}^{,}=\frac{1}{n}\sum_{i=1}^{n}\theta^{,}{\;}_{-i}=n\theta_{n}-\frac{n-1}{n}\sum_{i=1}^{n}\theta_{-i}$$

Variance of $\;\theta^{,}{\;}_{-i}$ :


$$s^{2}=\frac{1}{n\left(n-1\right)}\sum_{i=1}^{n}{\left(\theta_{-i}-{\bar{\theta }}^{,}\right)}^{2}$$

Based on the above method, 12 environmental factors that are relatively important to the geographical distribution of *A. rugosa* were screened out from 37 environmental factors (**Table 1**).

**Table 1:** Environmental factors involved in modeling.

type	Variable code	environmental factor	unit
climatic factor	bio3	Isothermal property	%
	bio5	Maximum temperature in hottest month	°C
	bio6	Minimum temperature in coldest month	°C
	bio8	The wettest quarterly average temperature	°C
	bio13	The wettest monthly precipitation	mm
	bio14	The driest monthly precipitation	mm
Topographic factor	elev	Altitude	m
	slope	Slope variability	%
Soil factor	t_cec_soil	Cation exchange capacity of topsoil	%
	t_caco3	Carbonate or lime content	%
	t_oc	Organic carbon content in topsoil	%
	t_bs	Basic saturation	%

### 2.2 Maxent model construction and optimization

Modeling by Maxent software. Randomly select 75% samples as training data set to model, and the remaining 25% distributed samples as test data set to verify the model. The maximum number of iterations is set to 10000, and the modeling is repeated for 10 times. Bootstrap is selected as the repetition type, and the distribution value is output in Logistic form. The accuracy of the model is evaluated by the value of AUC. The range of AUC value is 0. 5-1.0. The closer the AUC value is to 1, the more accurate the prediction is; 0. 5-0.7 means that the prediction effect is poor; 0. 8~0.9 means that the prediction effect is good; and 0.9-1.0 means that the prediction effect is very accurate (Wang et al., 2020).

The feature combination (FC) and regularization multiplier (RM) are adjusted by ENMeval data package in R software to optimize the model. There are five feature combinations of the Maxent model, namely Linear features, Quadratic features, Product features, Threshold features and Fragment features (Zhao et al., 2021a). In this study, the default parameters of Maxent software are RM = 1, FC =LQHPT; in order to optimize the Maxent model, RM is set to 0. 5 ~ 4, and every time it is increased by 0. 5, a total of 8 regulated frequency doubling is made. At the same time, six combinations of one or more characteristics are adopted: L, L and Q, H, L, Q, H and H, L, Q, H and P, L, Q, H, P and T. According to the permutation and combination, 48 parameter combinations are calculated. The combination of the above 48 parameters is tested, and the complexity of the model is tested according to the value of delta. AICc and the value of (auc.train-auc.diff.avg). The lower these two value are, the more accurate the prediction result of the model is.

### 2.3 Changes in spatial pattern of suitable distribution area for *A. rugosa*

Spatial units with a probability of species existence ≥ 0.374 are classified as suitable areas, while space units with a probability of species existence< 0.374 are classified as unsuitable areas. Using the reclassification tool in ArcGIS10.5 software binarize the potential geographical distribution data of *A. rugosa* under current and future climate change scenarios, and establish the presence/absence (0,1) matrix for the potential geographical distribution of *A. rugosa*. The suitable area is represented by a numeric value of “1” representing the existence of species, unsuitable area is represented by a numeric value of “0” representing the non-existence of species. Based on this matrix, further analysis was conducted on the spatial pattern changes of suitable areas of *A. rugosa* under current and future climate scenarios, and three types of changes in suitable areas were defined: newly added suitable areas, retreated suitable areas, and stable suitable areas. The spatial pattern changes of potential suitable areas under current and future climate change are defined as follows: matrix values 0 → 1 represents newly added suitable growth areas, 1 → 0 represents retreated suitable areas, and 1 → 1 represents stable suitable areas (Zhao et al., 2021b; Yao et al., 2023).

### 2.4 Niche analysis

In this study, the niche of *A. rugosa* in different periods and different climate modes was quantitatively analyzed. Based on the environmental factor layer and the distribution layer of *A. rugosa* in different periods and different climate modes, the niche was analyzed and visualized by using the “ecospat” package in R language (Yan et al., 2021), and the niche overlap index (percentage overlap index) was calculated, which was represented by the letter D, and its range was 0-1, indicating that the niche was from no overlap to complete overlap (Dan et al., 2008). Using the Niche breadth module in ENMTool, the niche width was calculated based on the potential distribution data onto *A. rugosa* in the current and future periods, with B1 representing the minimum niche width and B2 representing the maximum niche width.

### 2.5 Marxan model construction

Marxan model can be used to select the minimum cost area in the protection system. With the continuous improvement in the model, it has been widely used in the planning of land protection system (Xie et al., 2022). Taking the potential distribution area of *A. rugosa* in the current climate as the research object, the Marxan model was used to calculate the priority protected areas of *A. rugosa* in the current climate. Square Pu was used in the analysis, height and width were set to 25000. 1km^2^ as the research unit, using the Zonal Statistics as Table tool for ArcGIS, the distribution area of target species in each planning unit is counted, and the species distribution matrix is constructed. The protection target is set to 30% of the total habitat area and the SPF value is 100. (Li et al., 2019; Zhang et al., 2011), The boundary length modifier (BLM) of the model is the correction parameter of the boundary length of the protected area. By modifying the BLM, we can analyze the relationship between the cost and the total length and total area of the boundary, thus finding a balance point, and obtaining a more reasonable spatial distribution pattern of the protected area through calculation(Mou et al., 2021). The final model uses the model boundary modifier of 25,000. The model is iterated for 100 times to get the optimal solution to the planning unit.

### 2.6 Data processing

Using ArcGIS V10.5 software, the prediction results of Maxent model are transformed into raster data, and the values of the raster represent the survival probability of *A. rugosa* in this area. The natural discontinuous breakpoint grading method was used to divide the suitable areas into four grades: high suitable area (P≥0.7), moderate suitable area (0.57≤P< 0.7), low suitable area (0.374≤P<0.57) and unsuitable area (P<0.374). The data onto priority protected areas calculated by Marxan model are imported from ArcGIS, and the visualization of priority protected areas is realized by overlaying with the current distribution layer.

Using SDMTool data packet of R language, the centroid position of the suitable area of *A. rugosa* in current and future climate scenarios is calculated, and the migration direction of the spatial distribution of the suitable area of *A. rugosa* is reflected by the change of centroid position. Geosphere data packet of R language is used to calculate the centroid migration distance of *A. rugosa* in different climate scenarios.

## 3 Results

### 3.1 Optimal model and accuracy evaluation

**Figure 2:**
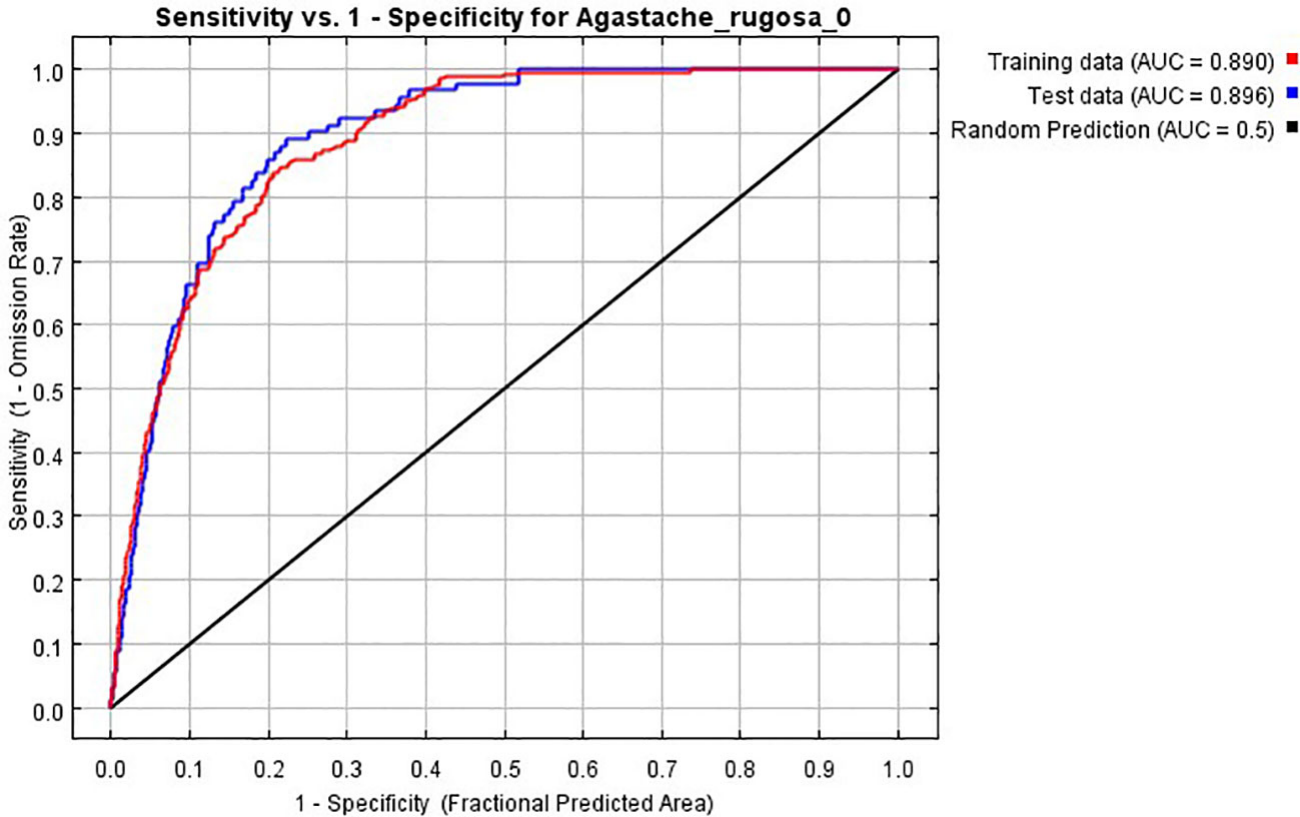
ROC response curve under Maxent model.

Based on 390 distribution points of *A. rugosa* and 12 environmental factors selected for modeling, the potential distribution area of *A. rugosa* was simulated by Maxent model. When the model is the default parameter, ΔAICc =11.88; when the model parameters are set to FC = L and RM = 0.5, ΔAICc = 0 (**Table 2**), which is the optimal model. Therefore, FC = L and RM = 0.5 are set as modeling parameters. Using the optimized parameters, the model was reconstructed to simulate the suitable area of *A. rugosa*, and the AUC value of simulated training for this parameter was 0.890 (**Figure 2**), which indicated that the prediction result was accurate.

**Table 2:** Evaluation results of Maxent model under different parameter settings.

Model evaluation	Feature combination	Regulated frequency doubling	Delta.AICc value	auc.train−auc.diff.avg
default	LQHPT	1	11.88	0.8305
optimize	L	0.5	0	0.6747

### 3.2 Distribution area of potential suitability of *A. rugosa* at present

**Figure 3:**
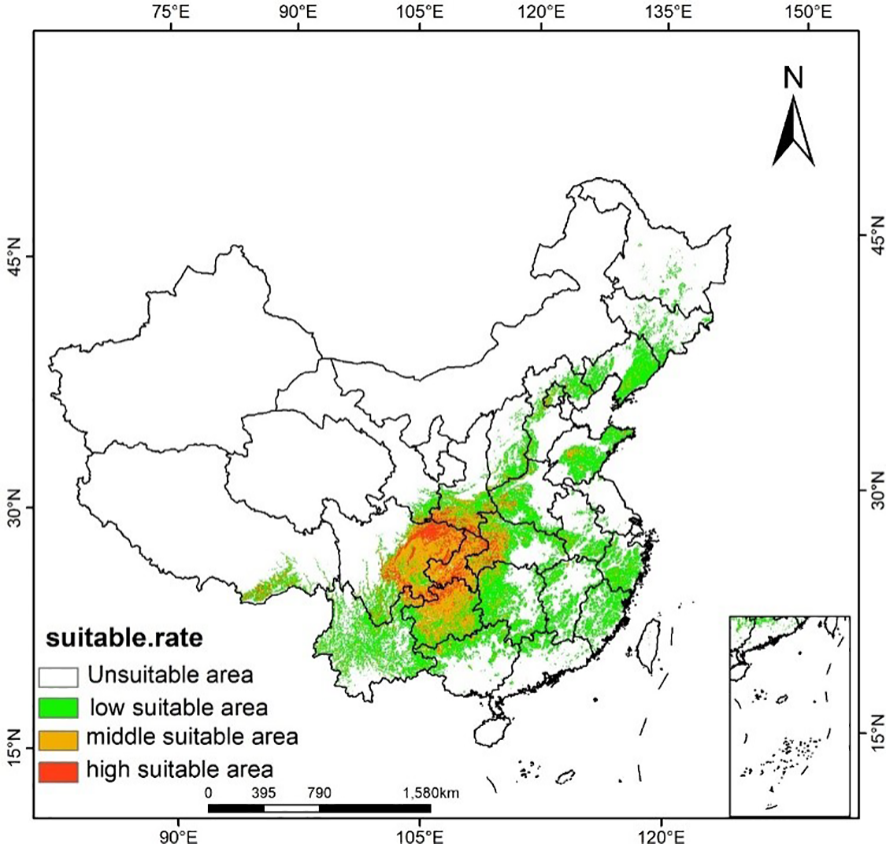
Potential Distribution Area of *A. rugosa* in China under current climate conditions.

Using ArcGIS software to classify the current distribution area of *A. rugosa*, and get the distribution map under the current climate conditions (**Figure 3**). As shown in **Figure 3**, the distribution area of *A. rugosa* is mainly concentrated on the eastern and central regions of China, covering Liaoning, Shanxi, Henan, Sichuan, Yunnan, Guizhou, Hunan, Hubei and other provinces. Among them, the highly suitable areas are mainly located in northeastern Sichuan, southwestern Shaanxi and western Hubei, while the moderately suitable areas are mainly located in eastern Sichuan and Guizhou. The simulation and prediction results are basically consistent with the geographical distribution data of *A. rugosa*, which further shows that the simulation and prediction results are accurate to some extent. According to the number of grids occupied by the suitable area, the area occupied by the suitable area can be calculated (**Table 3**). According to **Table 3**, the suitable area of *A. rugosa* in the current climate is 1,761,700 km^2^, accounting for 18.4% of China’s total land area, of which the highly suitable area is 138,700 km^2^, accounting for 4.7% of China’s total land area. It can be seen that the highly suitable area of *A. rugosa* in China is small and the growth range is concentrated.

**Table 3:** Suitable area of *A. rugosa* under different climatic scenarios (10,000 km^2^).

Climate change scenario	period	Low-suitability area	Moderately suitable area	Highly suitable area	Total suitable area
present	1970-2000	116.89	45.40	13.87	176.17
SSP1-2.6	2041-2060 2081-3000	153.29 156.89	59.26 57.80	26.88 25.12	239.44 239.81
SSP2-4.5	2041-2060 2081-3000	154.62 170.84	61.93 63.34	26.90 28.89	243.45 263.07
SSP5-8.5	2041-2060 2081-3000	185.71 191.60	73.26 93.88	30.67 43.06	289.64 328.54

### 3.3 Prediction of suitable areas of *A. rugosa* in future climate

The forecast result of Maxent model is converted into raster data, and the suitable area of *A. rugosa* under different climate scenarios is calculated. it can be seen that the suitable area of *A. rugosa* has increased compared with the current climate scenarios, but the growth rate is different, with the smallest growth rate under SSP1-2. 6, followed by SSP2-4.5 and SSP5-8.5 (**Table 3**). Taking 2090s as an example, it increased by 6.6%, 9.1% and 15.9% respectively.

**Figure 4:**
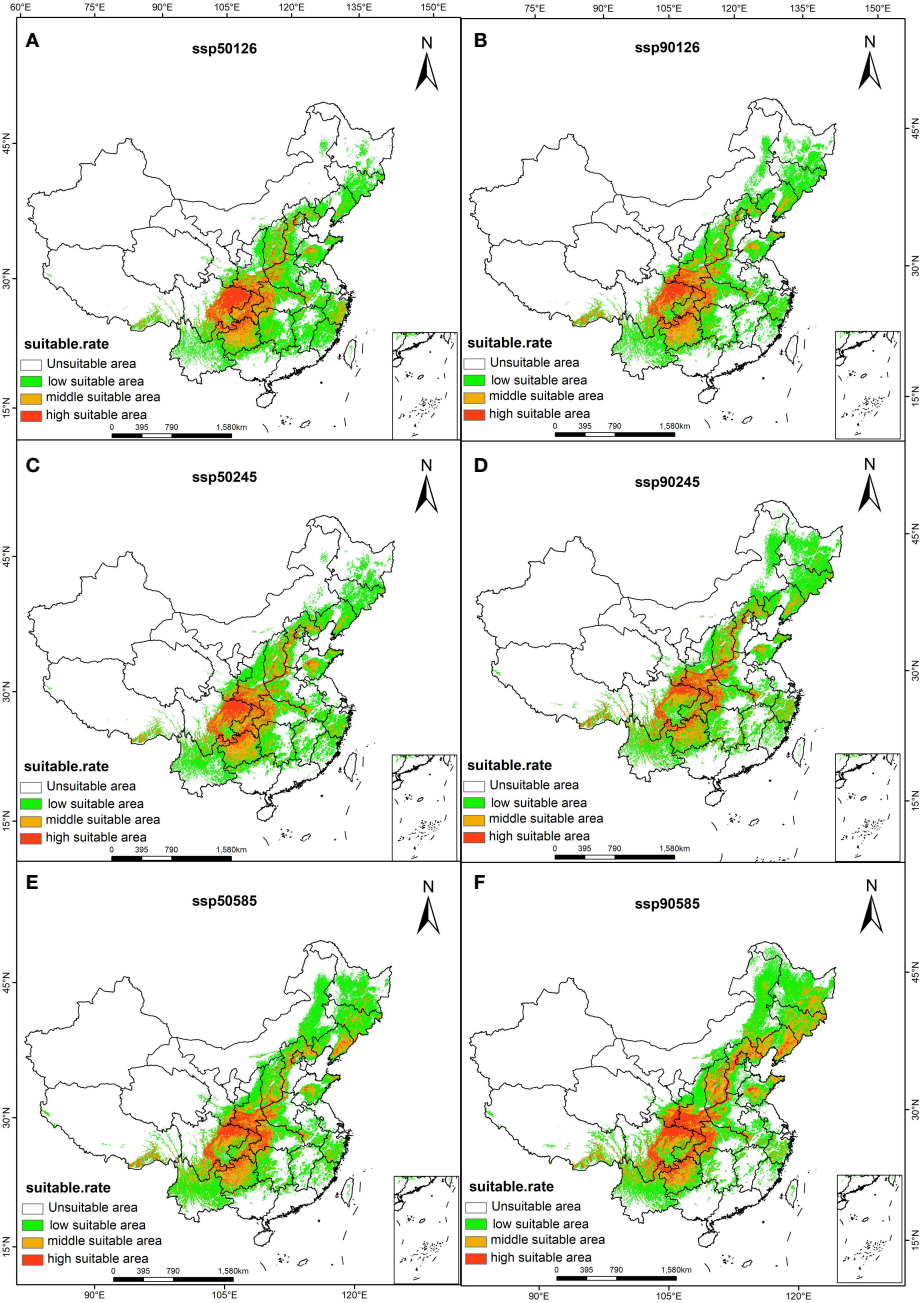
Suitable distribution areas of *A. rugosa* in ssp126 **(A, B)**, ssp245 **(C, D)**, ssp585 **(E, F)** scenarios in the 2050s **(A, C, E)** and 2090s **(B, D, F)**.

Using ArcGIS software to draw the suitable zoning map of *A. rugosa* in the future climate (**Figure 4**), by comparing the predicted suitable zones in 1950s and 1990s, we can see that there are differences in the response to *A. rugosa* to climate change in different scenarios. The change amplitude is the smallest in the SSP1-2.6 scenario, with an area of 239.4km^2^ in 2050s and 239.8km^2^ in 2090s, which is basically unchanged. However, under the climate scenarios of SSP2-4.5 and SSP5-8.5, the suitable area of *A. rugosa* increased greatly, increasing by 20 km^2^ and 39 km^2^ respectively. It can be seen that under the SSP1-2.6 scenario, the response to the suitable area of *A. rugosa* to climate change is not obvious, while under the SSP2-4.5 and SSP5-8.5 climate scenarios, the response of the suitable area of *A. rugosa* to climate change is more sensitive.

**Figure 5:**
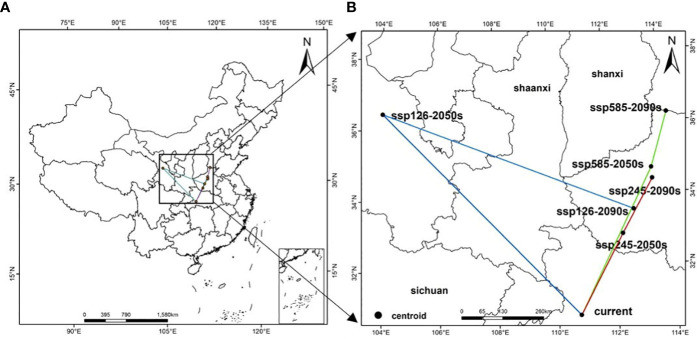
Geographical distribution changes of the centroid of the suitable area of *A. rugosa* under different climate scenarios [**(B)** is an enlargement of the part of **(A)**].

In terms of spatial pattern, there are some differences in the migration positions of the suitable areas of *A. rugosa* under different climate scenarios, but the overall migration trend is consistent, and it generally migrates to the northeast (**Figure 5**). At present, the center of mass of the suitable area of *A. rugosa* is located in Zigui County, Yichang City, Hubei Province (110.75°E, 30.70°N). When the climate scenario is SSP1-2.6-2090s, the center of mass of the suitable area of *A. rugosa* moves to the northeast, while the center of mass of the suitable area of *A. rugosa* is located in Lushan County, Pingdingshan City, Henan Province (112.8°E, 33.60°N) When the climate scenario is ssp 2-4.5-2090s, the center of mass of the suitable area moves to the northeast. At this time, the center of mass of the suitable area of *A. rugosa* is located in xinmi city, Zhengzhou City, Henan Province (113.50°E, 34.42°N), with a migration distance of 485,880 m; When the climate scenario is SSP5-8.5-2090s, the center of mass of the suitable area moves to the northeast. At this time, the center of mass of the suitable area of *A. rugosa* is located in Anyang County, Anyang City, Henan Province (114.20°E, 36.25°N), and the migration distance is 693,417 m. It can be seen that under the future climate change scenario, global warming and humidification will make the center of mass of the suitable area of *A. rugosa* in China move to the northeast as a whole, and the migration position will further expand upon the north.

### 3.4 Dynamic changes of suitable areas under different climatic scenarios at different periods

**Figure 6:**
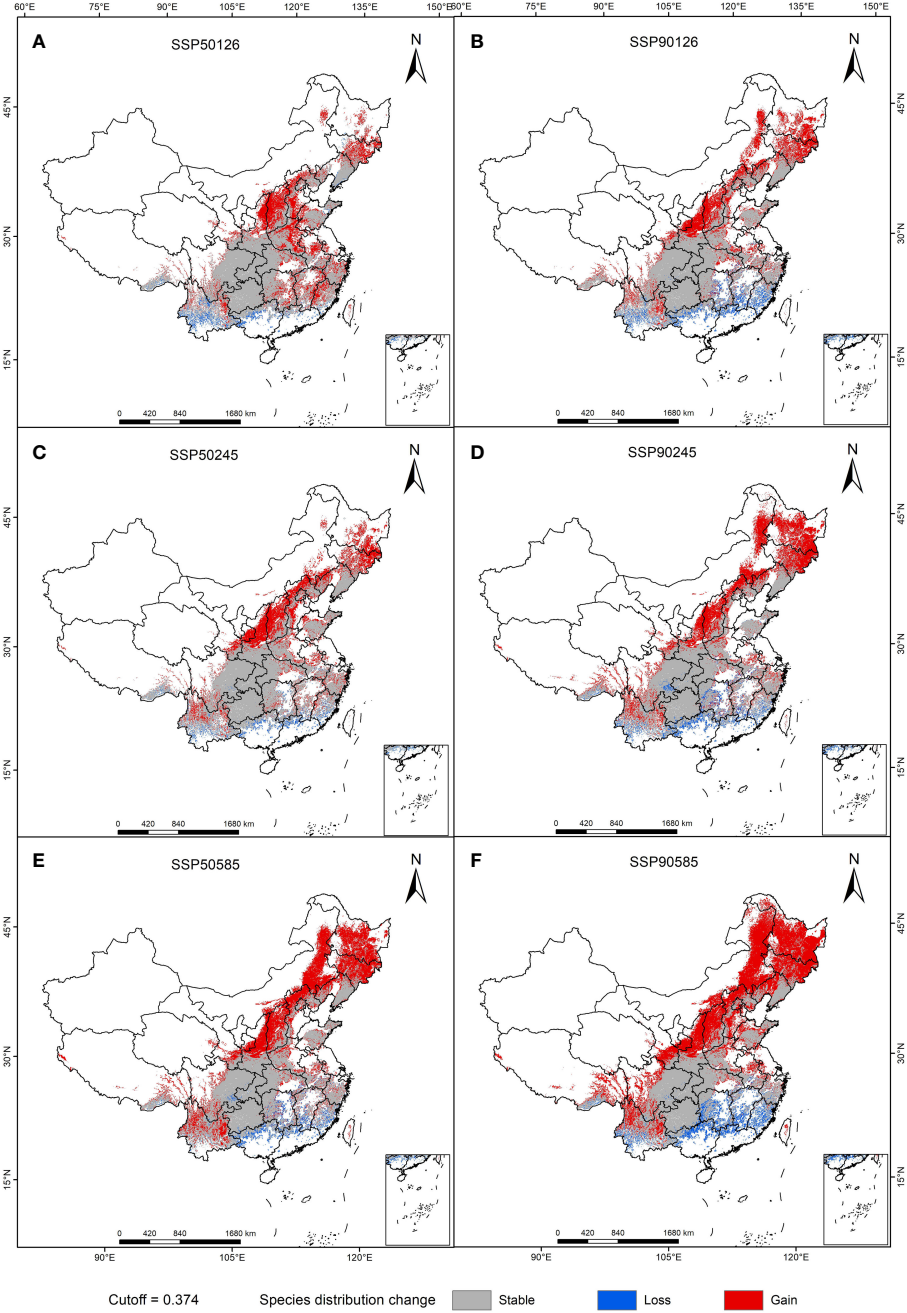
Spatial changes of geographical distribution of *A. rugosa* in ssp126 **(A, B)**, ssp245 **(C, D)**, ssp585 **(E, F)** scenarios in the 2050s **(A, C, E)** and 2090s **(B, D, F)**.

The distribution patterns of *A. rugosa* in different periods under different climate scenarios were compared and analyzed with the current period (**Table 4** and **Figure 6**), and the results showed that the suitable areas of *A. rugosa* in the future period and climate scenario were mainly increased. Among them, the expansion rate, retreat rate and stability rate of suitable area remained the highest in the scenario of 2081-2100 –SSP5-8.5: the area from unsuitable area to suitable area was 175.14km^2^, and the expansion rate was 99.42%. The area from suitable area to unsuitable area is 22.77km^2^, and the lost rate is 12.93%. The unchanged area is 153.4km^2^, and the stability rate is 86.49%. As can be seen from **Figure 6**, the expanded suitable areas are mainly concentrated in the northeast of China, such as Jilin, Hebei, Shanxi, Shaanxi and other provinces. The lost areas are mainly concentrated in the southeast of China, such as Fujian, Jiangxi, Hunan and other provinces. Generally speaking, the suitable area of *A. rugosa* changes with climate change, and the distribution pattern of *A. rugosa* in different periods under different climate scenarios has basically the same response to climate change. Among them, the change of the suitable area of *A. rugosa* is the most significant under the climate scenario of SSP5-8.5, and the change of the suitable area of *A. rugosa* is the least obvious under the climate scenario of SSP1-2.6.

**Table 4:** Changes in the distribution area of *A. rugosa* in different periods under different scenarios.

Period	Climate scenario	Habitat area (×10^4^ km^2^)	Loss (×10^4^ km^2^)	Stable (×10^4^ km^2^)	Gain (×10^4^ km^2^)	Species range change (%)	Percentage loss (%)	Percentage Gain (%)
Current		176.17						
2041-2060	SSP1-2.6	239.44	8.23	167.95	71.49	35.91	4.67	40.58
	SSP2-4.5	243.45	8.03	168.15	75.30	38.19	4.56	42.74
	SSP5-8.5	289.64	12.10	164.07	125.57	64.41	6.87	71.28
2081-2100	SSP1-2.6	239.81	15.46	160.72	79.09	36.12	8.77	44.89
	SSP2-4.5	263.07	14.88	161.29	101.78	49.32	8.45	57.77
	SSP5-8.5	328.54	22.77	153.40	175.14	86.49	12.93	99.42

### 3.5 Dominant climatic factors restricting the distribution of *A. rugosa*

The calculated habitat suitability and environmental factors are analyzed by knife cutting method. From the table of contribution rates of various environmental factors of the distribution of *A. rugosa* (**Table 5**), the top five environmental factors are the wettest monthly precipitation (59.1%), the lowest temperature in the coldest month (11.9%), altitude (9%), slope variability (6.6%) and the highest temperature in the hottest month (4.7). The top five environmental factors are the wettest monthly precipitation (42.5%), the lowest temperature in the coldest month (14.3%), the slope variability (9.8%), the highest temperature in the hottest month (9.2%) and the altitude (7.1%), accounting for 82.9% in total. Considering the contribution rate and important value, the dominant environmental factors restricting the distribution of *A. rugosa* are precipitation, temperature, altitude and slope.

**Table 5:** Contribution rate and important value of environmental factors.

environmental factor	Contribution rate%	Important value%
The wettest monthly precipitation	59.1	42.5
Minimum temperature in coldest month	11.9	14.3
altitude	9	7.1
Slope variability	6.6	9.8
Maximum temperature in hottest month	4.7	9.2
Isothermal property	3	6.4
Precipitation in the driest month	2.2	5.9
Basic saturation	1.3	1.5
Soil cation exchange capacity	0.8	0.3
Carbonate or lime content	0.6	1.1
Organic carbon content	0.6	1
The wettest quarterly average temperature	0.2	0.8

### 3.6 Environmental characteristics of *A. rugosa* suitable area

**Figure 7:**
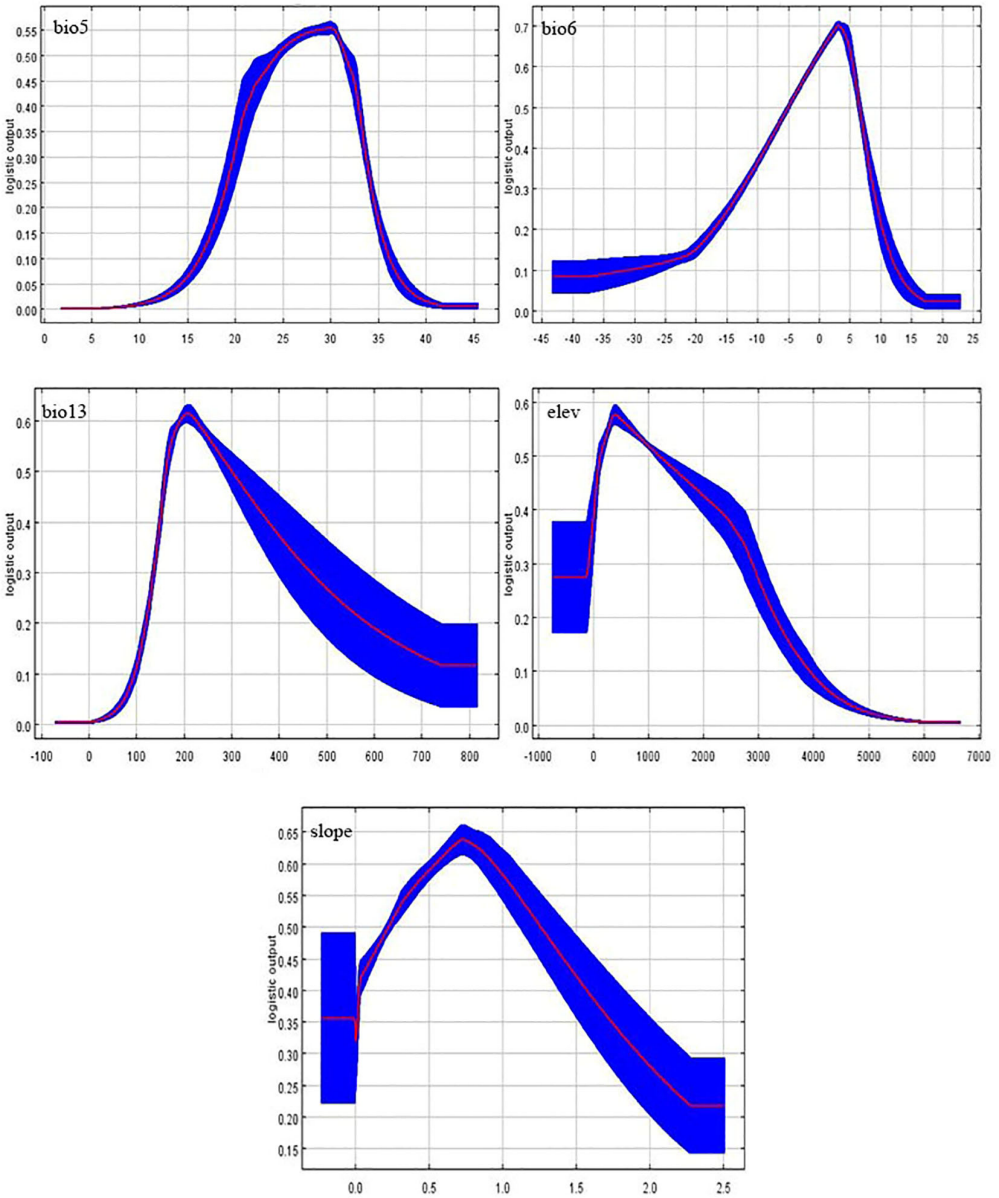
Single factor response curve of current climate.

The suitable area of species mainly depends on the dominant environmental factors. We can clearly understand the relationship between the distribution of *A. rugosa* and environmental factors by modeling 12 environmental factors and drawing the single factor response curve (**Figure 7**). It is generally believed that when the survival probability is greater than 0.5, the corresponding environmental factors are suitable for plant growth. It can be seen from **Figure 4** that when the wettest monthly precipitation (bio13) is less than 100ml, the survival probability of *A. rugosa* is less than 10%. After that, with the increase of precipitation, the survival probability of *A. rugosa* also increases. When the precipitation reaches 210ml, the survival probability reaches the peak; when the precipitation exceeds 210ml, the survival probability of *A. rugosa* decreases with the increase of precipitation. When the precipitation is between 160 ml and 300 ml, the survival probability of *A. rugosa* is greater than 0.5. Similarly, similar to the wettest monthly precipitation, there is an optimum range for the highest temperature in the hottest month, the lowest temperature in the coldest month, altitude and slope variability, and the survival probability of *A. rugosa* is less than 0.5 if it is too large or too small. Therefore, it can be inferred that under the current climate conditions, the wettest monthly precipitation in the suitable area of *A. rugosa* is 160ml-300ml, the highest temperature in the hottest month is 24°C-32°C, the lowest temperature in The coldest month is -5°C-7°C, the altitude is 100m-1200m, and the slope variability is 0.2×10^6^-1.3×10^6^. This is basically consistent with the environmental characteristics of the current potential distribution area of *A. rugosa* calculated in the result analysis of main environmental factors (**Table 6**), indicating that the prediction results are accurate.

**Table 6:** Result analysis of main environmental factors.

Environmental variable	Current	SSP1-2.6		SSP2-4.5		SSP5-8.5	
		2050s	2090s	2050s	2090s	2050s	2090s
Maximum temperature in hottest month	28.07	30.67	30.23	30.47	31.65	31.20	34.09
Minimum temperature in coldest month	-4.58	-2.49	-2.06	-1.59	-0.42	-1.35	0.33
Precipitation of wettest month	199.46	198.62	224.16	215.88	220.64	221.03	245.63
Altitude	851.37	851.37	851.37	851.37	851.37	851.37	851.37
Slope	3.0×10^5^	3.0×10^5^	3.0×10^5^	3.0×10^5^	3.0×10^5^	3.0×10^5^	3.0×10^5^
Suitability of species habitat	0.51	0.55	0.55	0.56	0.50	0.57	0.59

From the results analysis of main environmental factors (**Table 6**), it can be seen that the maximum temperature in the hottest month decreases from the passage of time for the SSP1-2.6 scenario, while the maximum temperature in the hottest month increases in the passage of time for the other two climate scenarios. The lowest temperature in the coldest month, the wettest monthly precipitation and the habitat suitability of species all show an increase in time for three climate scenarios. However, the altitude and slope variability remain basically unchanged. It can be inferred that in the future climate scenario, the temperature in the suitable area of *A. rugosa* will increase compared with the current overall, and the precipitation will increase.

### 3.7 Analysis of niche differences of *A. rugosa* under different climate models

**Figure 8:**
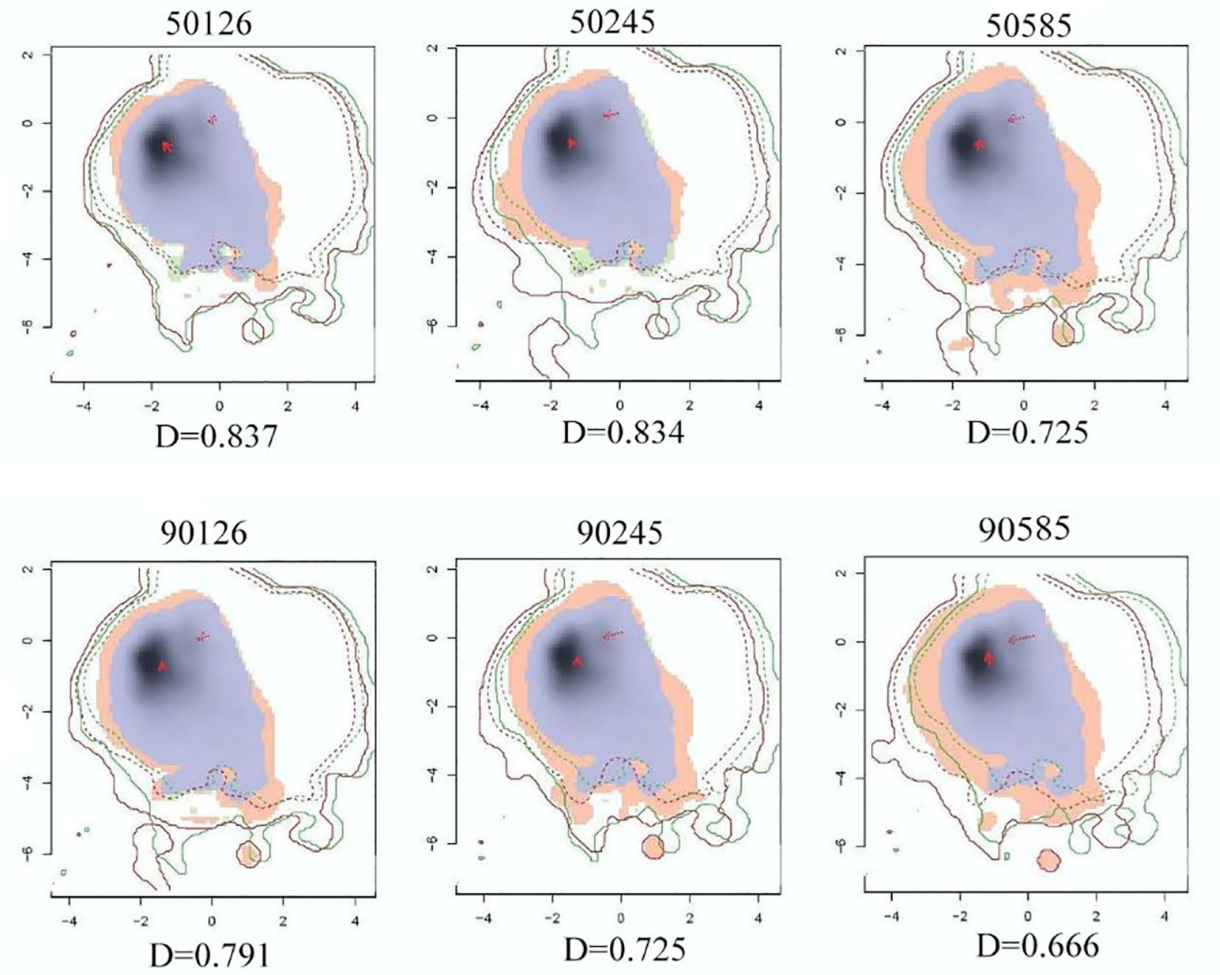
Niche differences of A.rugosa in different climatic backgrounds in the future.

The niche space of *A. rugosa* under different climate models was visualized. As shown in **Figure 8**, the niche overlap between *A. rugosa* in different climatic backgrounds is relatively large (the maximum niche overlap D_50126 = _0.837; The minimum niche overlap D_90585 = _0.666), so there is no significant niche differentiation. In addition, in the same period, with the increase of radiation intensity, the niche overlap of *A. rugosa* tended to decrease gradually, among which the niche overlap in the climate scenario of SSP5-8.5 was significantly lower than that in the other two climate scenarios. Compared with 2050s, the niche overlap of *A. rugosa* in 2090s decreased to different degrees under each radiation intensity. This shows that the resources that can be used together in the future and the current period are reduced.

ENMTools software package was used to calculate the niche width of *A. rugosa* in different climate backgrounds. As shown in **Table 1** supplementary materials, the maximum value of B1 is 0.557 and the minimum value is 0.436; the maximum value of B2 is 0.967, and the minimum value is 0.951. Therefore, there is no obvious difference between B1 and B2 in each period, which indicates that *A. rugosa* is more inclined to be a generalized species. In addition, compared with the current period, B1 and B2 have increased in other climate scenarios, which indicates that all kinds of resources that *A. rugosa* can use in the future climate scenarios have increased and are widely distributed, and have strong adaptability to the future environment.

### 3.8 Priority protected areas of *A. rugosa* under current climate conditions

**Figure 9:**
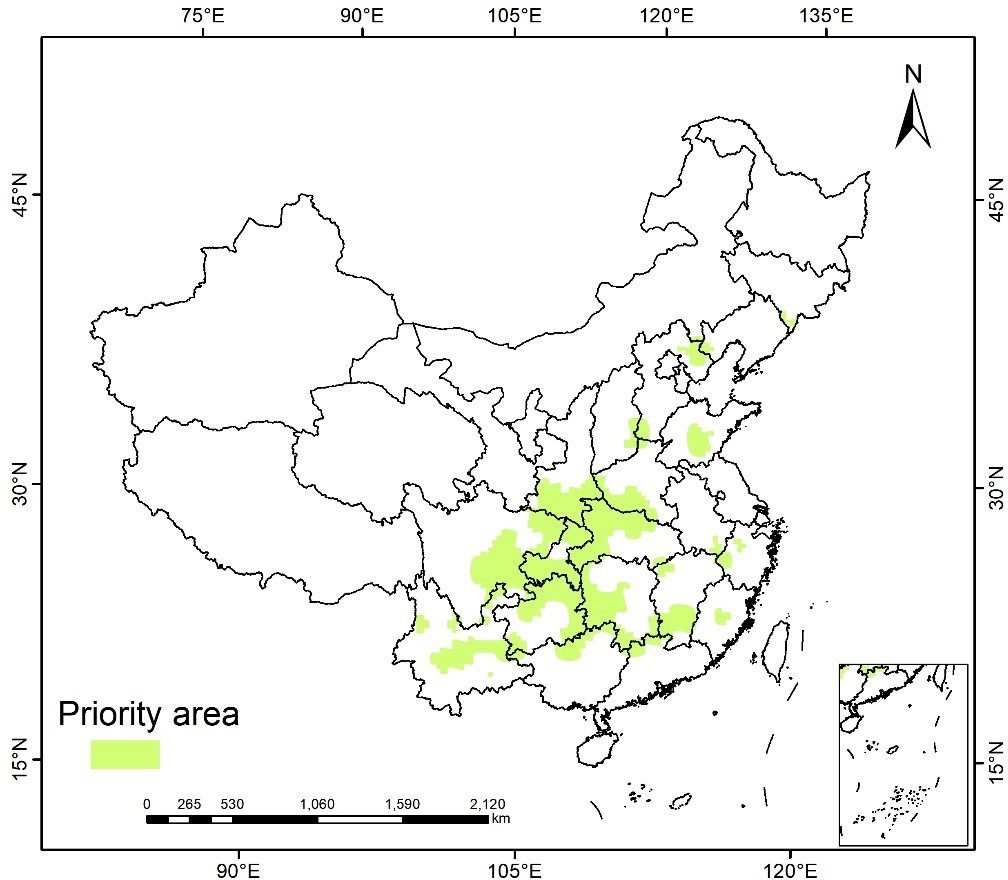
priority protected areas of *A. rugosa* in China predicted by MARXAN model.

The priority protected areas of *A. rugosa* were calculated by marxan model, and the results were imported from ArcGIS software to generate a system protection plan of *A. rugosa* as the main protection target. As shown in **Figure 9**, the priority protected areas of this species are concentrated on Chongqing, eastern Sichuan, southern Guizhou, western Hunan and Hubei, and southwestern Shaanxi, which is basically consistent with the highly suitable areas of *A. rugosa* predicted by Maxent model, indicating that the prediction results are accurate. In addition, the priority protected area of *A. rugosa* occupies a small proportion of the land area, and the distribution is concentrated, which is conducive to the formulation of targeted protection and management.

## 4 Discussion

### 4.1 Model simulation evaluation

Based on environmental variables such as climate, topography and soil factors, this study applies ENMeval data package to optimize the model. This method limits the background data onto the area corresponding to the calibration position, so that the potential geographical distribution area simulated by Maxent covers the current distribution point. This method allows the model parameters to be adjusted to improve the performance of the Maxent model, and its accuracy can be measured by improving the fitting degree between the prediction results and the actual distribution area and by visual inspection of the geographical prediction map (Guo et al., 2018). The Maxent model with optimized parameters can effectively reduce the complexity of the model, improve the fitting degree between the predicted results and the actual situation, predict the species distribution effectively, and the response curve obviously becomes smooth, and it is close to the normal distribution curve, which conforms to Shelford’s tolerance law (Phillips et al., 2017; Li et al., 2018; Ouyang et al., 2019). Many related studies have confirmed that the concentration of sample size and distribution points of species distribution data will significantly affect the accuracy of species model simulation results. In general, with the increase of sample size, the simulation accuracy of species distribution model increases, and the increasing range gradually decreases until it no longer increases, and finally tends to reach the maximum accuracy of the model (Chen et al., 2012). Too concentrated distribution points will increase the over-fitting of environmental deviation caused by spatial autocorrelation of distribution points, which will have a certain impact on the simulation results of the model (Yao et al., 2023). In this study, a total of 505 distribution points of *A. rugosa* were collected, and the correlation between the distribution point data and environmental variables was analyzed and screened, and finally 390 distribution point data were obtained, which solved the inaccurate modeling results caused by the small sample size and strong multicollinearity among environmental factors. Some studies show that the change of research scale will lead to the change of background data, and then affect the model construction (Ji et al., 2019; Ma and Li, 2023). In this study, the potential distribution area of *A. rugosa* is predicted at the national scale. If the study is conducted at a larger or smaller scale, the selection of background data points should be effectively compared with the occurrence points of species, rather than simply randomly extracting background points from the research scope. However, how to choose a reasonable background data point is still worth further study. At present, there is no unified standard for the classification of the grade in the suitable area, and the selection of the threshold directly affects the area of the suitable area, which may lead to a big difference between the divided range of the highly suitable area and the actual distribution range, so it is necessary to select an appropriate threshold for the classification and compare it with the actual distribution range (Wang et al., 2020).

### 4.2 Environmental factors restricting the distribution of *A. rugosa*

Under the current climate conditions, the main environmental factors affecting the distribution of *A. rugosa* are precipitation and temperature, followed by topographic factors, and soil factors have the lowest influence on *A. rugosa*. Based on the response curves of various factors, the wettest monthly precipitation in the suitable area of *A. rugosa* can reach 300ml, the highest temperature can reach 32°C and the lowest temperature can reach -5°C, which is consistent with the biological characteristics of *A. rugosa* that it likes high temperature, humidity and cold resistance of roots. Although hydrothermal conditions play a major role in the potential geographical distribution pattern of *A. rugosa* in China, the constraints of topographic factors and soil factors cannot be ignored. The contribution rate of environmental factors shows that many factors jointly affect the potential geographical distribution of *A. rugosa*, such as temperature, moisture, altitude, slope and so on.

### 4.3 Changes in the potential geographical distribution of *A. rugosa*

Under different climate scenarios in the future, the potential distribution of *A. rugosa* in China is quite different from that at present, indicating that future climate warming will have a certain impact on the geographical distribution of *A. rugosa*. Compared with the current climate scenario, the area and spatial pattern of the highly suitable area of *A. rugosa* have changed significantly. In 2050s, the area of highly suitable areas under the three climate scenarios did not change much, but in 2090s, the area of highly suitable areas under the three climate scenarios showed an increasing trend in turn, indicating that the growth rate of the distribution area of *A. rugosa* accelerated with the passage of time, so it can be inferred that the global warming situation is also gradually intensifying. The response of the spatial pattern of the suitable area to climate change is consistent on the whole, that is, with the intensification of climate warming, the overall migration range of the spatial position of the suitable area of *A. rugosa* becomes larger. The migration trend of *A. rugosa* is consistent with the geographical distribution of temperate tree species migrating to high latitudes under future global warming (Zhang et al., 2019). The size of niche overlap reflects the similarity of plant utilization of resources. The large niche overlap shows that they have similar ecological requirements, resource utilization and biological characteristics under certain circumstances (Yuan et al., 2021). The decrease of resources that can be used together in the future and the current period indicates that the geographical distribution area of *A. rugosa* has changed under the influence of global climate change, which is consistent with the overall migration of the center of mass of the suitable area of *A. rugosa* predicted by Maxent model to the northeast. Compared with the current period, the niche width of *A. rugosa* in the future climate scenario has increased, indicating that the area of its suitable area has increased under the influence of global change, which is consistent with the prediction results of Maxent model and proves the accuracy of Maxent model. The prediction of the future suitable area of *A. rugosa* is based on the assumption that the soil and topographic factors have not changed for 70 years(Zhang et al., 2018). The research results are only valid at the national and provincial levels, and cannot be applied to local microclimate. Agastache species all have similar morphological and physiological characteristics, and its geographical distribution may also have some similarities in response to climate change, so this model has certain guiding significance for the prediction of distribution areas of other species of Agastache in future climate scenarios.

### 4.4 Priority protected areas of *A. rugosa*

From the perspective of nature reserves, the systematic protection zoning for *A. rugosa* is explored. The results show that the priority protected areas are concentrated on Chongqing, eastern Sichuan, southern Guizhou, western Hunan and Hubei, and southwestern Shaanxi. These areas are mostly warm and humid mountainous areas with high altitude and terrain, good natural conditions such as light, moisture and temperature, which are consistent with the growth habits of most medicinal plants and are very suitable for the growth of Chinese herbal medicines (Liu et al., 2021). The establishment of protected areas in mountainous areas has less human interference and is more conducive to plant protection. At the same time, the priority protected areas suitable for the growth of *A. rugosa* have certain guiding significance for the industrialization development of *A. rugosa* in the future, and the development of *A. rugosa* planting base can also be taken as the primary task to promote the local economic development.

## 5 Conclusions

*A. rugosa* is mainly distributed in the eastern and central regions of China, covering Liaoning, Shanxi, Guizhou, Hubei and other provinces at present. Hydrothermal conditions are the main environmental factors that affect the distribution of *A. rugosa*. With the intensification of global warming, the suitable areas for *A. rugosa* will increase in the future climate conditions, otherwise a few areas will shrink back and migrate to high latitudes as a whole. The priority protected areas for *A. rugosa* are mainly distributed in Chongqing, eastern Sichuan, southern Guizhou, western Hunan and Hubei and southwestern Shaanxi, which are basically consistent with the highly suitable areas predicted by MaxEnt model. Therefore, it is inevitable for planning the nature reserve to take into account the impact of future climate change and moderately move to the northeast on the basis of the current forecast areas. In this study, when predicting the distribution of Agastache rugosa in the future, other data are unchanged except the climate data, which may cause some deviations. Therefore, the results of this study should be verified in the local environment, and at the same time, considering the microclimate conditions, changeable soil and topographic conditions, the model input should be calibrated to obtain more accurate predictions and better explain the output. In addition, the results of this study are only valid at the national and provincial levels, and cannot be applied to local microclimates.

## Data availability statement

Publicly available datasets were analyzed in this study. This data can be found here: ;https://datadryad.org/stash/dashboard.

## Author contributions

YW: Conceptualization, methodology, software, formal analysis, investigation and writing - original draft. RZ: Writing - review and editing, supervision, methodology and resources. XYZ: Resources. XLZ: Software, writing, funding acquisition. GZ: Software, methodology and formal analysis. FZ: Funding acquisition, supervision, writing, review and editing. All authors contributed to the article and approved the submitted version.
